# Early Canities among Undergraduate Medical Students of a Medical College: A Descriptive Cross-sectional Study

**DOI:** 10.31729/jnma.7961

**Published:** 2023-03-31

**Authors:** Sonam Chaudhary, Narayan Mahotra

**Affiliations:** 1 Department of Clinical Physiology, Maharajgunj Medical Campus, Maharajgunj, Kathmandu, Nepal

**Keywords:** *epidemiology*, *hair colour*, *medical students*, *physiology*

## Abstract

**Introduction:**

Early canities are the premature greying of hair before the age of 25 years in Asians. The condition is a matter of concern for young adults aesthetically. This study aimed to find out the prevalence of early canities among undergraduate medical students of a medical college.

**Methods:**

A descriptive cross-sectional study was carried out from 1 December 2021 to 30 June 2022 among undergraduate medical students at a medical college. The study was conducted after receiving ethical approval from the Institutional Review Committee [Reference number: 146(6-11) C-2 078/079]. The participants with ages less than 25 years without a history of vitiligo, intake of chemotherapeutic drugs, progeria, pangeria and recent dyeing of hair were enrolled. A convenience sampling method was used. Point estimate and 95% Confidence Interval were calculated.

**Results:**

Out of 235 students, 95 (40.42%) (34.15-46.69, 95% Confidence Interval) had early canities. The most prevalent premature greying was grade I early canities i.e.79 (83.15%) of participants. Among the participants with early canities, 56 (58.94%) were male, 41 (43.15%) had a positive family history for early canities, 67 (70.52%) had normal body mass index and 38 (40%) had O+ve blood group.

**Conclusions:**

The prevalence of early canities among undergraduate medical students was lower than in other studies done in similar settings. The grade I early canities was seen more among the participants with premature greying of hair.

## INTRODUCTION

Early canities are premature greying of hair which occurs regardless of gender or race. Hair is said to grey prematurely only if it happens before the age of 20 years in Whites, before 25 years in Asians, and before 30 years in Africans.^1^

The early canities have significant adverse effects on the appearance, self-esteem, and socio-cultural acceptance of the affected individual.^2^ It has been found that the early exhaustion of melanocyte reservoir or some defect in melanocyte activation/migration in combination with different environmental factors, inflammation or psychological stress may lead to early canities.^3^ Several types of research are being conducted worldwide to find the prevalence and risk factors associated with early canities which is lacking in the context of Nepal.

This study aimed to find the prevalence of early canities among undergraduate medical students in a medical college.

## METHODS

A descriptive cross-sectional study was conducted in the Department of Clinical Physiology of Maharajgunj Medical Campus from 1 December 2021 to 30 June 2022. The MBBS students from preclinical and clinical years were enrolled after getting ethical approval from Institutional Review Committee [Reference number: 146(6-11 )C-2078/079]. The undergraduate medical students of both sexes with Nepalese origin and aged less than 25 years were included after verbal informed consent. The participants who had dyeing of hair grey or white colour or suffered from vitiligo or had symptoms of premature ageing like progeria or pangeria, or had a history of intake of chemotherapeutic drugs were excluded. A convenience sampling method was used. The sample size was calculated by using following formula:


$$\begin{array}{ll} \text{n} & ={\text{Z}}^{2}\times \frac{\text{p}\times \text{q}}{{\text{e}}^{\text{2}}}\\ & =1.96^{2}\times \frac{0.712\times 0.288}{0.06^{2}}\\ & =219 \end{array}$$

Where,

n = minimum required sample size
Z = 1.96 at 95% Confidence Interval (CI)
p = prevalence of early canities, 71.2%^4^
q = 1-p
e = margin of error, 6%

The calculated minimum required sample size was 219. However, a total of 235 undergraduate medical students were included in the study.

The study variables included were sex, body mass index (BMI), blood group (ABO and Rh system) and familial predisposition of premature greying among the participants. The BMI was calculated with the ratio of weight in kilograms (kg) by height in square meters (m^2^) and was further classified according to Asia Pacific Guidelines as underweight if BMI was <18.5 kg/m^2^, normal weight if BMI was between 18.5-22.9 kg/m^2^, overweight if BMI was between 23-24.9 kg/m^2^ and obese if BMI was more than 25 kg/m.^2,5^ The semistructured questionnaire was used for the diagnosis and classification of early canities as done among the Korean population.^6^ According to this method, the early canities were graded into five categories i.e., grade I (less than 20% greying in total hair), grade II (20-40% greying), grade III (40-60% greying), grade IV (60-80% greying), and grade V (more than 80% greying).^6^

Data were entered in Microsoft Excel 2013 and analysed using IBM SPSS Statistics version 16.0. Point estimate and 95% CI were calculated.

## RESULTS

**Figure 1:**
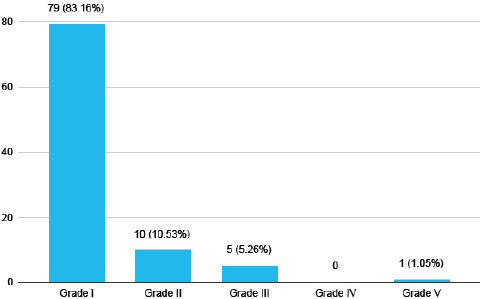
Grading of early canities among medical students with early canities (n= 95).

Among 235 undergraduate medical students, 95 (40.42%) (34.15-46.69, 95% CI) had early canities. Grade I early canities were seen in 79 (83.15%) students followed by grade II seen in 10 (10.52%) (Figure 1).

The number of male students with early canities was 56 (58.94%). The maximum number of participants with early canities were non-smokers 93 (97.89%), 67 (70.52%) had normal BMI, and 54 (56.84%) had a negative family history of early canities (Table 1).

**Table 1:** Characteristics of medical students with early canities (n= 95).

Characteristics	n (%)
**Sex**	
Male	56 (58.94)
Female	39(41.05)
Positive family history	41 (43.15)
**BMI**	
Underweight	13 (13.68)
Normal weight	45 (47.36)
Overweight	22 (23.15)
Obese	15 (15.78)
**Blood group**	
A+ve	21(22.10)
B+ve	28 (29.47)
O+ve	38 (40)
AB+ve	4(4.21)
A-ve	1 (1.05)
B-ve	-
O-ve	2 (2.1)
AB-ve	1 (1.05)

## DISCUSSION

Our study has found the prevalence of early canities among medical students between 18-25 years age was 40%. The early canities have been less studied in our context. A similar study was done in Karnataka, India among 12-19 years age group participants have found the prevalence rate of early canities was 1.2%.^7^ Similar study conducted in the Korean population has found the prevalence of early canities was 25.3%.^8^ However a study conducted among 80 medical students in Indonesia has found that the prevalence of early canities was 71.2%.^4^

Grade I early canities were reported among 83.15% of the participants with early canities in this study. A study conducted among the Korean population has also shown a higher prevalence of grade I early canities i.e. 85.9%.^6^ However, there has been no universally accepted method for the grading of early canities. The grading of early canities was done as mild (<50 grey hair), moderate (50-100 grey hair) and severe (>100 grey hair) in an Indian study which reported a 65.7% distribution of moderate early canities among the participants who had premature greying of hair.^7^

A retrospective study among patients with early canities in North India has shown that gender was equally distributed.^9^ Male-to-female ratio in another study among the Indian population was 1:1.1.^7^ Our study has found that the male-to-female ratio in participants with early canities was 1.43:1. The higher distribution of early canities among males was also seen in a Nigerian study with a male: female ratio of 1.46:1.^10^

It was reported that 90.1-42.6% cases with early canities in Indian studies had a positive family history for early canities.^7,9^ This positive family history of premature greying was seen among 39% of early canities participants among college students in Indonesia.^11^ Our study has found that positive family history was 43.15% among the participants with premature greying of hair.

Similarly, the obesity distribution was 15.78% among the participants with early canities in our study. However, obesity has also been linked as a risk factor for early canities in the Korean population.^8^

There is a lack of data regarding the condition of premature greying in the Nepalese population for comparison. Similarly, due to the lack of universally accepted methods for grading early canities, there is a paucity of literature to compare and contrast the severity of early canities among the participants. Similarly, this study was conducted in a single centre and the participants were enrolled using a convenience sampling method so the findings might not be generalizable to a larger population. Further research with a multicenter study could be done for the estimation of the prevalence.

## CONCLUSIONS

The prevalence of early canities among undergraduate medical students was lower than in other studies done in similar settings. The condition has however aesthetic concern among young adults. The proper risk factors associated with the condition should be identified with a higher study design for its possible management. This study can be considered as a preliminary resource for further exploration of the topic among the Nepalese population.
